# Hepatoprotective effect of the solvent extracts of *Viola canescens* Wall. ex. Roxb. against CCl_4_ induced toxicity through antioxidant and membrane stabilizing activity

**DOI:** 10.1186/s12906-016-1537-7

**Published:** 2017-01-05

**Authors:** Abdullah, Mir Azam Khan, Waqar Ahmad, Manzoor Ahmad, Mohammad Nisar

**Affiliations:** 1 Department of Pharmacy, University of Malakand, Chakdara, Pakistan; 2 Department of Chemistry, University of Malakand, Chakdara, Pakistan; 3 Department of Botany, University of Malakand, Chakdara, Pakistan

**Keywords:** *Viola canescens*, Hepatoprotective, CCl_4_ induced hepatotoxicity

## Abstract

**Background:**

*Viola canescens* Wall. ex. Roxb. exhibits analgesic, antimalarial and antispasmodic activities. It is used folklorically for the treatment of liver diseases, hypertension, malaria and cancer. The current study investigates phytochemical constituents, antioxidant and hepatoprotective activity of solvent extracts of whole plant of *Viola canescens.*

**Methods:**

Phytochemicals, acute toxicity study and antioxidant activity of *Viola canescens* methanolic extract (VCME), ethyl acetate fraction (EAF), and partially purified EAF (90% EAF and combination of 80% EAF + 20% methanol fraction (EAF + Me) was carried out. Hepatoprotective activity of VCME, EAF (200 and 400 mg/kg body weight) and partially purified EAF (50 mg/kg body weight) was investigated in carbon tetrachloride (CCl_4_) intoxicated BALB/c mice for 7 days. Membrane stabilization effect was determined by hypotonic solution induced hemolysis while DNA ladder assay was carried out by polyacrylamide gel electrophoresis.

**Results:**

Phytochemical screening of VCME showed the presence of alkaloids, phenols, flavonoids, saponins, carbohydrates, tannins and triterpenes. VCME, EAF (at 200 and 400 mg/kg body weight) and partially purified EAF (90% EAF and EAF + Me) at 50 mg/kg body weight significantly reduced the level of ALT, ALP, total bilirubin and restored the level of serum protein in comparison to CCl_4_ treated group. A significant reduction in malondialdehyde (MDA) and elevation in catalase (CAT) and superoxide dismutase (SOD) level was observed in extract treated animals as compared to CCl_4_ (*p* < 0.05). The IC_50_ values in membrane stabilization potential for VCME, EAF and sodium salicylate were 3.7 ± 0.11, 3.4 ± 0.15 and 3.2 ± 0.09 mg/ml, respectively. Similarly, CCl_4_ induced degradation of DNA was counteracted by VCME and EAF. The liver biopsy of mice treated with the solvent extracts showed remarkable restoration of normal histological archeitecture.

**Conclusions:**

*Viola canescens* showed significant hepatoprotective potential due to its antioxidant and membrane stabilization effect.

## Background

Liver exposure to drugs, toxic chemicals and environmental pollutants results in the formation of reactive oxygen species (ROS) that are retained responsible, at least in part, for hepatitis, cirrhosis, hepatic cancer and many other disorders [1]. The carbon tetrachloride (CCl_4_) induced hepatotoxicity in rodents resembles the viral hepatotoxicity in humans which renders it a suitable model for hepatoprotective drugs development [2]. In liver, CCl_4_ results in formation of free radicals such as trichloromethyl and trichloromethylperoxyl radicals that bind to macromolecules such as DNA, lipids and proteins [3]. These free radicals extract hydrogen atoms from the lipid membrane of hepatocytes to form lipid hydroperoxides which ultimately leads to liver necrosis [4, 5]. The increased level of lipid hydroperoxides and free radicals cause reduction in the level of antioxidant enzymes, along with oxidative DNA damage, genetic mutation, chromosomal alteration and low CYP2E1 activity. Inbuilt antioxidant defense system of hepatocytes consists of catalase, superoxide dismutase, glutathione system, ascorbic acid and tocopherol that provide protection against free radical mediated damage [6, 7]. Oxidative stress, occuring due to imbalance between antioxidant defence system and ROS production, is involved in the pathophysiological changes associated with various liver disorders such as hepatitis, hepatocellular carcinoma and liver cirrhosis [8]. Therefore an insight into the role of oxidative stress and antioxidants in liver ailments may help in the development of natural and more effective drugs. Now days, antioxidants from natural sources are increasingly being used in liver diseases and about 50% of drugs used in liver diseases are, either, natural products or their derivatives [9].

The currently used synthetic drugs for the treatment of hepatic disorders are inadequate and have severe adverse reaction [10, 11]. Silymarin, a hepatoprotective drug from *Silybum marianum*, consist of silybin, isosilybin, silydianin, silychristin and taxifolin, is associated with the drawback of poor solubility and low bioavailability [12–16]. There is an intense need to search for and develop more efficient hepatoprotective drugs with better solubility, high bioavailability, economical and safe.

Medicinal plants are enriched with polyphenols and flavonoids that have the potential to terminate free radicals, improve the level of antioxidant enzymes, modulate gene expression and hence provide protection against ROS [17, 18]. Family Violaceae comprise of 20 genera and 800 species while in Pakistan one genus (Viola) and 17 species of this family are reported [19]. Compounds isolated from Viola include flavonoids, phenylpropanoids, terpenoids, amides, sterols, essential oils, saccharides, aromatic acids and cyclotides. Viola has exhibited a number of activities such as antioxidant, antibacterial, anti-inflammatory, immunomodulatory, antimalarial, anticancer, insecticidal, anti-HIV, anxiolytic, anticonvulsant, cytotoxic, hepatoprotective and lung protective activity [20]. *Viola odorata* exhibited hepatoprotective activity against paracetamol induced hepatotoxicity due to the presence of flavonoids (isorhamnetin and luteolin) [21–23]. Similarly, during preliminary phytochemical investigation of the *Viola canescens*, polyphenols and flavonoids, beside saponins, triterpenes and alkaloids [24–26] were found to be present in appreciable amount dictating its possible role as an effective hepatoprotectant. *V. canescens* has exhibited antimalarial, analgesic and antispasmodic activity [27, 28]. This plant has also been reported for its use in liver disorders [29, 30], in addition to hypertension [31], eczema, malaria, rheumatism, gastric acidity, dysentery, respiratory tract problems, pyrexia, epilepsy and cancer [32] in traditional medicines. Keeping in view the folkloric use beside flavonoids and polyphenolic contents of *Viola canescens* and their well established role in combating oxidative stress as well as hepatotoxicity, the current study was designed to investigate antioxidant and hepatoprotective potential of *Viola canescens* as well as to study the underlying mechanism responsible for hepatoprotection.

## Methods

### Chemicals

DPPH, acrylamide, bisacrylamide, thiobarbituric acid, tetramethoxy propane (Sigma Aldrich), methanol, *n-*hexane, chloroform, ethyl acetate (commercial grade), diclofenac sodium (Novartis), phenobarbital sodium (Swan Pharma, Islamabad Pakistan), Tween 80, gallic acid (BDH), formalin (Scharlu), xylene, trichloroacetic acid, carbon tetrachloride, ascorbic acid, Folin Ciocalteau reagent, TLC cards, silica (Merck), silymarin (Zhejiang Chemicals Hangzhou, China), enzymatic kits such as ALT, ALP, total bilirubin, total protein (Vitro scientific, Germany), DNA ladder, ethidium bromide (Thermo Fisher Scientific).

### Plant material

*Viola canescens* was collected from Dir Upper, Khyber Pakhtunkhwa Pakistan in March 2013 and authenticated by Professor Dr. Muhammad Ibrar, from Department of Botany University of Peshawar. The specimen was submitted to the herbarium, University of Malakand under reference number H.UOM.BG. 209. The fresh plant was cleaned, dried in shade, powdered, weighed (3 kg), subsequently soaked in 80% methanol (10 L, three times) for 15 days and then filtered [33]. The filtrate was concentrated at 40 °C in rotary evaporator to obtain a semisolid residue of methanolic extract of *Viola canescens* (VCME) having percentage yield of 9.3% (280 g). VCME was further subjected to fractionation resulting in *n*-hexane fraction (NHF) with yield of 2.33% (70 g), chloroform fraction (CF) 0.83% (25 g), ethyl acetate fraction (EAF) 1.6% (48 g), butanol fraction (BTF) 1.06% (32 g) and aqueous fraction (AQF) 2.8% (84 g).

### Partial purification of ethyl acetate fraction

EAF was subjected to partial purification over silica gel through column chromatography. The optimum solvent system for column chromatography was determined by TLC. EAF was loaded into glass column by slurry method and eluted with 4 l of each of these solvents; 100% *n*-hexane, followed by *n*-hexane-ethyl acetate in ratio of 90:10, 80:20, 70:30, 50:50, 40:60, 30:70, 10:90 (90% EAF) and then 100% ethyl acetate. Successive fractions were collected and concentrated in rotary evaporator at 40 °C. Afterwards, the column was eluted with methanol: ethyl acetate in ratio of 5:95, 10:90, 15:85, 20:80 and finally washed with 100% methanol [34, 35]. TLC was carried out with hexane: ethyl acetate (3:7). Fractions having same retardation factor (R_f_) were compiled for further analysis.

### Phytochemical investigation

VCME was investigated by qualitative tests for carbohydrates, saponins, steroids, flavonoids, triterpenes, tannins, alkaloids, proteins, fixed oils and fats according to procedures described by Harborne [36].

### Total phenolic content

Calibration curve for gallic acid was constructed at 20–100 μg/ml. Afterwards, 1 ml extract in distilled water (0.1 g/100 ml) was added to 5 ml Folin Ciocalteau reagent (1: 10 aqua) and 4 ml sodium carbonate (7.5%), mixed and incubated at room temperature for 30 min followed by absorbance determination at 765 nm by spectrophotometer (Thermoscientific, USA) against blank. Total phenolic content (GAE/g) was calculated from the calibration curve [37].

### Total flavonoid content

Total flavonoid content was determined as mentioned in literature [38]. Extracts (0.3 ml), methanol (3.4 ml, 30%) aluminium chloride (0.15 ml, 0.3 M) and sodium nitrite (0.15 ml, 0.5 M) were mixed in a test tube. After 5 min sodium hydroxide (1 ml, 1 M) was added to the mixture followed by absorbance determination at 506 nm by spectrophotometer (Thermoscientific, USA) against blank. Calibration curve for standard (Quercitin) was constructed at 5–100 mg/l. The quantity of total flavonoids was determined from the standard curve as mg of quercitin equivalent per g (QE/g).

### Antioxidant activity

#### DPPH assay

The antioxidant activity of the solvent extracts of the plant and the standard was determined by DPPH method [39]. Methanolic solution of plant extracts and ascorbic acid were prepared at 31.25, 62.5, 125, 250 and 500 μg/ml. Methanolic solution of DPPH (20 μg/ml, 1 ml) was mixed with 1 ml of standard and sample solution. The resultant solutions were kept in dark for 30 min at room temperature and absorbance was measured at 517 nm by UV-visible spectrophotometer.

#### Hydrogen peroxide assay

H_2_O_2_ scavenging activity was performed as mentioned in literature [40]. H_2_O_2_ solution (0.6 ml, 40 mM) in phosphate buffer (pH 7.4) was mixed with extracts (31.25 to 500 μg/ml). After 10 min, absorbance of H_2_O_2_ was determined against blank at 230 nm. Percent H_2_O_2_ scavenging and IC_50_ of extracts and standard (alpha tocopherol) were calculated.

#### Experimental animals

BALB/c mice of 20–30 g were obtained from NIH Islamabad, Pakistan and housed in the stainless steel cages in the animal house, University of Malakand maintained on standard diet, free access to water and food, with 12 h dark and light cycle.

#### Acute toxicity study

Acute toxicity study was carried out according to the guidelines of Organization for Economic Cooperation and Development 423 [41]. Healthy mice were randomly assigned into five groups (*n* = 6) and fasted overnight prior to experiment. Group I was administered Tween 80. Extracts were dissolved in Tween 80 and administered to group II-V as a single dose of 250, 500, 1000 and 2000 mg/kg body weight of VCME, EAF, 90% EAF and EAF + Me (80% EAF + 20% Methanol) fraction p.o., followed by observation for toxicity and behavioral changes at 30 min and then 2,4, 8, 24 and 48 h.

### Hepatoprotective activity

#### Direct method

BALB/c mice were divided into nine groups (*n* = 6) [42]. Group I received liquid paraffin 0.8 ml/kg, i.p, which was used as vehicle for CCl_4_. Group II mice were treated with CCl_4_ i.p (30%, 0.8 ml/kg). Group III was administered CCl_4_ (0.8 ml/kg i.p) plus silymarin 100 mg/kg p.o. Similarly, group IV-VII were orally administered with VCME and EAF at 200 and 400 mg/kg respectively for 7 consecutive days. While groups VIII-IX were administered partially purified EAF (90% EAF and EAF + Me (80% ethyl acetate + 20% methanol) at 50 mg/kg. All animals, except group I, were administered CCl_4_ at 0.8 ml/kg i.p for 7 days. Blood was collected from all mice by cardiac puncture after 24 h of the last dose administration, under diethyl ether anesthesia, followed by laparatomy and liver removal. All the animals were weighed before and after experimental period for changes in body weight.

#### Indirect method (Phenobarbital-induced sleep model)

Phenobarbital induced sleeping method was performed as mentioned in literature [43]. The experiment was designed as mentioned before. On the 7^th^ day, all the mice were administered phenobarbital sodium (40 mg/kg i.p) followed by determination of the time between the loss and regain of righting reflex (sleeping time).

### Assessment of hepatoprotection

#### Biochemical investigation

Collected blood was centrifuged at 1000 rpm for 10 min for serum separation that was subsequently used for estimation of ALT, ALP, total bilirubin and total protein as per established methods using commercially available enzymatic kits (Vitro scientific, Germany).

#### Estimation of antioxidant enzymes

The liver was removed carefully, weighed (1 g) and homogenized in 10 ml of ice-cold phosphate buffer (50 mM, pH 7.4).

Activity of catalase activity was determined according to already mentioned protocol [44]. Briefly, 3 ml of reaction mixture consist of 1.9 ml buffer (pH 7.0), 1 ml of the H_2_O_2_ and 0.1 ml of liver homogenate. The activity was determined from the change in absorbance at 240 nm in UV–visible spectrophotometer. The activity was presented as unit of H_2_O_2_/mg of tissue.

For superoxide dismutase activity determination, sodium carbonate buffer (2.8 ml, 0.05 mM) and 0.1 ml of liver homogenate were incubated at 30 °C for 45 min. Afterwards, adrenaline solution (10 μL, 9 mM) was added and the absorbance was determined at 480 nm against blank. The results were reported as unit of SOD activity/mg of tissue [45].

#### Lipid peroxidation

The liver homogenate (0.1 ml) was added to trichloroacetic acid (2.0 ml, 20%), mixed and centrifuged at 4000 rpm for 20 min. The obtained supernatant (2 ml) was added to thiobarbituric acid reagent (2 ml). Standard (tetramethoxypropane) (5–20 nmoles) and blank were also prepared in the same way. The mixtures were incubated on water bath at 100 °C for 20 min followed by absorbance determination at 532 nm in UV–visible spectrophotometer. The lipid peroxide contents were reported as moles MDA per 100 mg of protein [46].

### Membrane stabilization potential

Membrane stabilization potential of VCME and EAF was determined by hypotonic solution induced human erythrocyte haemolytic assay as per previously published protocol [47]. Healthy human volunteers who have not taken NSAIDs prior to the experiment were used in the study. Collected blood (5 ml) was centrifuged at 2500 rpm for 5 min followed by removal of supernatant. Isotonic buffer was used to wash the cell suspension until the supernatant appeared clear. Erythrocyte suspension (40% v/v) was made with isotonic buffer and 50 μl of the cell suspension was mixed with 1.0 ml of hypotonic buffer and 100 μl of the solvent extracts. After 20 min incubation at room temperature the samples were centrifuged (5000 rpm, 5 min), supernatant was separated and its absorbance determined at 540 nm. Sodium salicylate was used as reference. Percent inhibition of erythrocyte haemolysis by the extracts and standard was calculated as

$$ \%\ \mathrm{Inhibition}\ \mathrm{of}\ \mathrm{haemolysis} = {\mathrm{OD}}_{\mathrm{c}}\hbox{--}\ {\mathrm{OD}}_{\mathrm{s}}/{\mathrm{OD}}_{\mathrm{c}} \times 100 $$

Where OD_c_ is absorbance of control and OD_s_ is absorbance of sample. The concentration needed to inhibit 50% of erythrocytes lysis as compare to the control (IC_50_) was calculated from the dose response curves.

### DNA isolation and ladder assay

DNA isolation from hepatic tissue was carried out according to previously reported method [48]. The tissue was homogenized in 1 ml of lysis buffer [0.15 M NaCl, 20 mM Tris-Cl (pH 7.5), 1 mM EDTA, 1 mM EGTA, 1% Triton X-100 and 25 mM disodium pyrophosphate] at 37 °C for 1 h. Afterwards, 0.4 ml of saturated sodium chloride was added to cell lysates followed by incubation on ice for 5 min and centrifugation at 3000 rpm for 30 min. Chilled ethanol was used to precipitate the DNA that was separated through centrifugation. DNA washing was carried out with 70% ethanol followed by drying and then resuspended in Tris-EDTA buffer followed by quantification. Same amount of sample DNA and standard DNA ladder were loaded on 30% polyacrylamide gel containing ethidium bromide. Electrophoresis was carried out for 90 min at 100 volts, and DNA was observed under UV-transilluminator and photographed.

### Histopathological study

The liver was fixed in 10% neutral buffered formalin for 24 h, followed by dehydration in different concentrations of alcohol and xylene, embedded in paraffin wax, subsequently sectioned with microtome (4 μm), stained with hematoxylin and eosin (H&E) and observed under light microscope and photomicrographs were taken [49]. The liver sections were scored according to previously reported protocol [50].

### Statistical analysis

Statistical analyses were performed by SPSS version 16 (SPSS Inc. Chicago IL, USA). All the data are reported as a mean of 6 animals per group ± SEM. Statistical analyses were conducted by one way ANOVA followed by *post hoc* Tukey test for multiple comparisons. All the values of *p* < 0.05 were considered significant.

## Results

### Phytochemical investigation

The results of phytochemical investigation show the presence of carbohydrates, alkaloids, phenols, flavonoids, saponins, tannins and triterpenes with no proteins, fixed oils, fats and steroids.

### Total phenol content

Total phenol content of methanol extract and different fractions is presented in Table 1. EAF had highest phenol content (107.5 ± 0.14 mg GAE/g) followed by CF (92.5 ± 0.09 mg GAE/g), VCME (87.5 ± 0.13), BTF (77.5 ± 0.06), AQF (52.5 ± 0.02) while the least quantity was found in NHF (27.5 ± 0.04 mg GAE/g). The descending order of phenol content was EAF > CF > VCME > BTF > AQF > NHF.

**Table 1:** Total phenolic and flavonoid content of *Viola canescens*

Sample	Total phenol content (mg GAE/g)	Total flavonoid content (mg QE/g)
VCME	87.5 ± 0.06	59 ± 0.13
NHF	27.5 ± 0.04	22 ± 0.16
CF	92.5 ± 0.02	76 ± 0.09
EAF	107.5 ± 0.14	80 ± 0.25
BTF	77.5 ± 0.09	72 ± 0.18
AQF	52.5 ± 0.13	45 ± 0.06

### Total flavonoid content

Total flavonoids content is shown in Table 1. EAF had maximum flavonoids content (80 ± 0.25 mg QE/g) followed by CF (76 ± 0.09), BTF (72 ± 0.18), VCME (59 ± 0.13), AQF (45 ± 0.06), while NHF had the least amount (22 ± 0.04 mg QE/g). The descending order of flavonoid content was EAF > CF > BTF > VCME > AQF > NHF.

### Antioxidant activity

#### DPPH assay

**Fig. 1:**
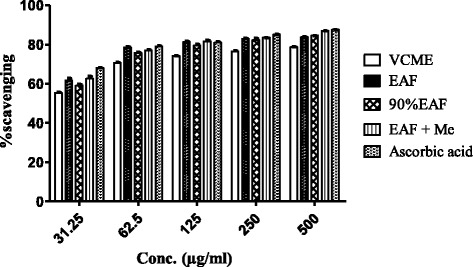
DPPH assay of *Viola canescens*

EAF + Me exhibited highest DPPH scavenging effect (86.89 ± 0.16%, IC_50_ = 12 μg/ml), followed by 90% EAF (84.5 ± 0.04%, IC_50_ = 14 μg/ml), while EAF had DPPH radical scavenging activity (83.7 ± 0.26%, IC_50_ = 15 μg/ml), and for VCME (78.6 ± 0.10, IC_50_ = 26 μg/ml) (Fig. 1).

#### Hydrogen peroxide scavenging potential

**Fig. 2:**
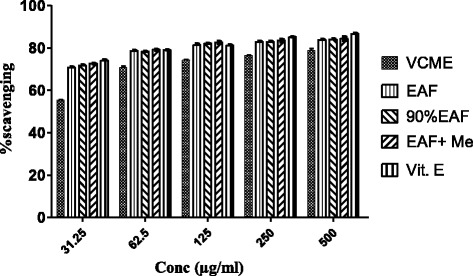
Hydrogen peroxide scavenging assay of *Viola canescens*

The partially purified EAF exhibited better scavenging action as compared to other fractions (Fig. 2). IC_50_ for H_2_O_2_ scavenging was 4, 5, 7, 25, 15, μg/ml for Ascorbic acid, EAF + Me, 90% EAF, VCME and EAF respectively.

#### Acute toxicity

Oral administration of the solvent extracts up to 2000 mg/kg body weight did not produce toxicity as evident from lack of diarrhea, drowsiness, convulsions, writhing, respiratory distress and mortality.

#### Hepatoprotective activity

The serum level of ALP, ALT and total bilirubin was significantly elevated and total protein declined by CCl_4_ administration (Table 2). VCME caused significant reduction in level of ALT, ALP, total bilirubin and elevation in total protein level in comparison to the CCl_4_ treated group (*p* < 0.05). Furthermore, EAF reduced the ALT level from 195.6 ± 1.49 to 59.66 ± 0.88 at 400 mg/kg as compare to silymarin (*p* > 0.05). Group administered with silymarin 100 mg/kg p.o decreased ALT level from 195.6 ± 1.49 to 54.5 ± 1.17 as compared to normal (*p* > 0.05). Likewise, among the partially purified fractions, EAF + Me exhibited optimum hepatoprotection at 50 mg/kg as compared to silymarin in terms of decline of ALT from 195.67 ± 1.49 to 50.66 ± 1.76 and total bilirubin from 2.74 ± 0.077 to 0.7 ± 0.05 (*p* > 0.05). While 90% EAF resulted in decrease of ALT from 195.67 ± 1.49 to 76.33 ± 6.35 (*p* < 0.05) and total bilirubin from 2.74 ± 0.077 to 0.813 ± 0.06 (*p* > 0.05) as compared to silymarin. Similarly, total protein level was improved by administration of VCME, EAF and more significantly elevated by partially purified EAF as shown in Table 2.

**Table 2:** Effect on liver biomarkers

	ALT (U/L)	ALP (U/L)	T.B (mg/dl)	T.P (g/dl)
Normal	48.16 ± 1.6	176.8 ± 2.1	0.76 ± 0.01	5.09 ± 0.18
CCl_4_	195.60 ± 1.4	315.3 ± 1.7	2.73 ± 0.07	3.91 ± 0.47
Silymarin	54.50 ± 1.17	190.6 ± 2.2	0.89 ± 0.01	4.77 ± 0.37
VCME 200	93.00 ± 1.15***	250.8 ± 2.7***	1.32 ± 0.02***	4.18 ± 0.21
VCME 400	85.30 ± 1.11***	239.1 ± 1.2***	1.23 ± 0.01***	4.25 ± 0.14
EAF 200	74.16 ± 1.22***	208.6 ± 1.2***	1.06 ± 0.03***	4.4 ± 0.12
EAF 400	59.66 ± 0.88 ns	195.3 ± 1.6 ns	0.99 ± 0.01 ns	4.61 ± 0.18***
90% EAF	76.33 ± 0.73***	193.0 ± 1.08 ns	0.83 ± 0.08 ns	4.75 ± 0.42***
EAF+ Me	50.67 ± 0.44 ns	187.0 ± 0.68 ns	0.70 ± 0.05 ns	4.78 ± 0.25***

***p < 0.05 as compare to standard

ns *p* > 0.05 as compare to standard

### Antioxidant enzymes

The activities of catalase and superoxide dismutase were significantly reduced by CCl_4_ as compared to corresponding control group. Their level was improved by VCME, EAF and partially purified EAF (Table 3).

**Table 3:** Effect on antioxidant parameters

	Catalase (U/mg protein)	SOD (U/mg protein)	Mol MDA/100 mg protein
Normal	40.24 ± 0.73***	53.42 ± 1.12***	14.8 ± 0.72***
CCl_4_	15.87 ± 0.24	18.3 ± 0.95	43.5 ± 0.45
Silymarin	38.7 ± 0.4	52.65 ± 2.1	16.8 ± 0.06
VCME 200	22.2 ± 0.11	29.81 ± 2.34	34.3 ± 0.13
VCME 400	27.5 ± 0.15	32.45 ± 0.55	26.1 ± 0.2
EAF 200	34.4 ± 0.9	40.37 ± 0.66	17.5 ± 0.42***
EAF 400	39.76 ± 0.53***	51.88 ± 1.18***	16.2 ± 0.55***
90% EAF	39.93 ± 0.16***	52.12 ± 0.55***	15.4 ± 0.09***
EAF+ Me	40.17 ± 0.08***	53.3 ± 0.28***	15.1 ± 0.1***

***Values are significantly different as compared to CCl_4_ *p* > 0.05

### Lipid peroxidation

MDA level was enhanced by CCl_4_ administration as compare to control. Co-administration of VCME and ethyl acetate fractions to CCl_4_-treated mice resulted in partial recovery of MDA. The high dose of extracts (400 mg/kg) was more effective as compare to low dose (200 mg/kg) (Table 3). Moreover, partially purified EAF resulted in near to normal level of MDA.

### Effect on body and liver weight

Significant loss in body weight (−5.5 ± 6.845%) was observed in the CCl_4_ intoxicated group as compared to normal during the study period. There was gain in body weight due to administration of EAF (4.9 ± 4.4%) and partially purified ethyl acetate fractions, EAF + Me (6.5 ± 0.782%) and 90% EAF (4.6 ± 0.488%) which is comparable to silymarin (*p *> 0.05). Moreover, CCl_4_ administration resulted in gain of liver weight which was successfully countered by EAF and partially purified EAF (90% EAF and EAF + Me). There was insignificant difference between liver weights of mice administered with EAF as compare to silymarin (*p *> 0.05) as shown in Table 4.

**Table 4:** Effect on body and liver weight

	Initial body weight (g)	Final body weight (g)	Percent change	Liver weight (g)	Liver index (%)
Normal	23.91 ± 1.22	26.26 ± 0.80	9.8	1.735 ± 0.16	6.60
CCl_4_	24.04 ± 0.80	22.71 ± 0.48	−5.5	1.84 ± 0.175	8.10
Silymarin	24.48 ± 0.34	25.42 ± 0.30	3.83	1.69 ± 0.045	6.65
VCME 200	22.97 ± 0.64	22.49 ± 0.70	−2.08	1.64 ± 0.19	7.30
VCME 400	22.16 ± 0.53	22.51 ± 0.11	1.57	1.66 ± 0.42	7.38
EAF 200	25.80 ± 0.84	26.92 ± 1.10	0.46	1.70 ± 0.14	6.31
EAF 400	23.55 ± 0.22	24.72 ± 0.33	4.9	1.72 ± 0.40	6.95
90% EAF	24.230 ± 0.27	26.86 ± 0.375	4.6	1.72 ± 0.19	6.4
EAF+ Me	23.87 ± 1.68	25.43 ± 1.88	6.5	1.73 ± 0.11	6.8

### Phenobarbital induced sleeping time

CCl_4_ induced prolongation of phenobarbital sleeping time (PST) as compare to normal group; however administration of VCME, EAF reduced sleeping time as compare to silymarin (Tables 5 and 6). Furthermore, 90% EAF and EAF + ME had more efficient reduction of PST that signifies its better hepatoprotective activity.

**Table 5:** Effect on phenobarbital induced sleeping time in mice

S. No	Group	Sleeping duration (minutes)	% recovery
1	Control	85 ± 2.26	---
2	CCl_4_	130 ± 3.88	----
3	Silymarin 100	98 ± 1.36***	71.11
4	VCME 200	115 ± 0.92***	33.33
5	VCME 400	108 ± 0.88	48.88
6	EAF 200	101 ± 1.46	64.44
7	EAF 400	97 ± 0.93***	73.33
8	90% EAF	98 ± 1.06***	71.11
9	EAF+ Me	96 ± 1.15***	75.55

Values are means ± S.E.M, *n* = 6

***p < 0.001 as compare to CCl_4_ group

**Table 6:** Histopathological effects of *Viola canescens*

Groups/Observations	Fatty changes	Centrilobular necrosis	Piecemeal necrosis	Congestion in sinusoids	Lymphocyte infiltration
Control	-	-	-	-	-
CCl_4_	+++	++++	+++	+++	+++
Silymarin	+	-	-	-	+
VCME	++	+	+	+	+
EAF	+	-	-	-	+
90% EAF	-	-	-	+	+
EAF + Me	-	-	-	+	-

-, absent; +, mild; ++, moderate; +++, severe; ++++, extremely severe

### Membrane stabilization potential

**Fig. 3:**
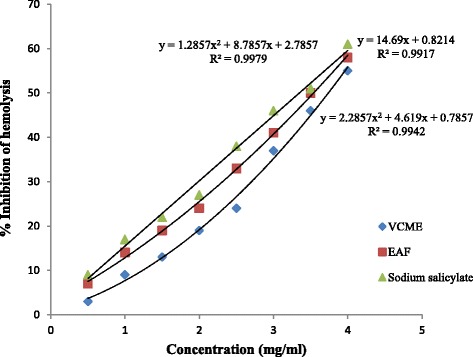
Membrane stabilization potential

VCME and EAF resulted in stabilization of RBC membrane. The membrane stabilizing effect of EAF was higher as compare to VCME which is in agreement with biochemical study. The IC_50_ values for VCME, EAF and sodium salicylate were 3.7 ± 0.11, 3.4 ± 0.15 and 3.2 ± 0.09 mg/ml (Fig. 3).

### DNA ladder assay

**Fig. 4:**
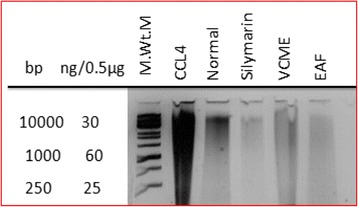
Effect on DNA by polyacrylamide gel electrophoresis

Protective effect of VCME and EAF on CCl_4_ induced DNA damage in the liver tissue of mice is shown by DNA ladder assay (Fig. 4). Extensive DNA breaking in hepatic tissue was observed in mice administered with CCl_4_. Concurrent administration of silymarin, VCME and EAF protected the DNA from damage showing hepatoprotective effect of *Viola canescens.*

### Histopathological observations

**Fig. 5:**
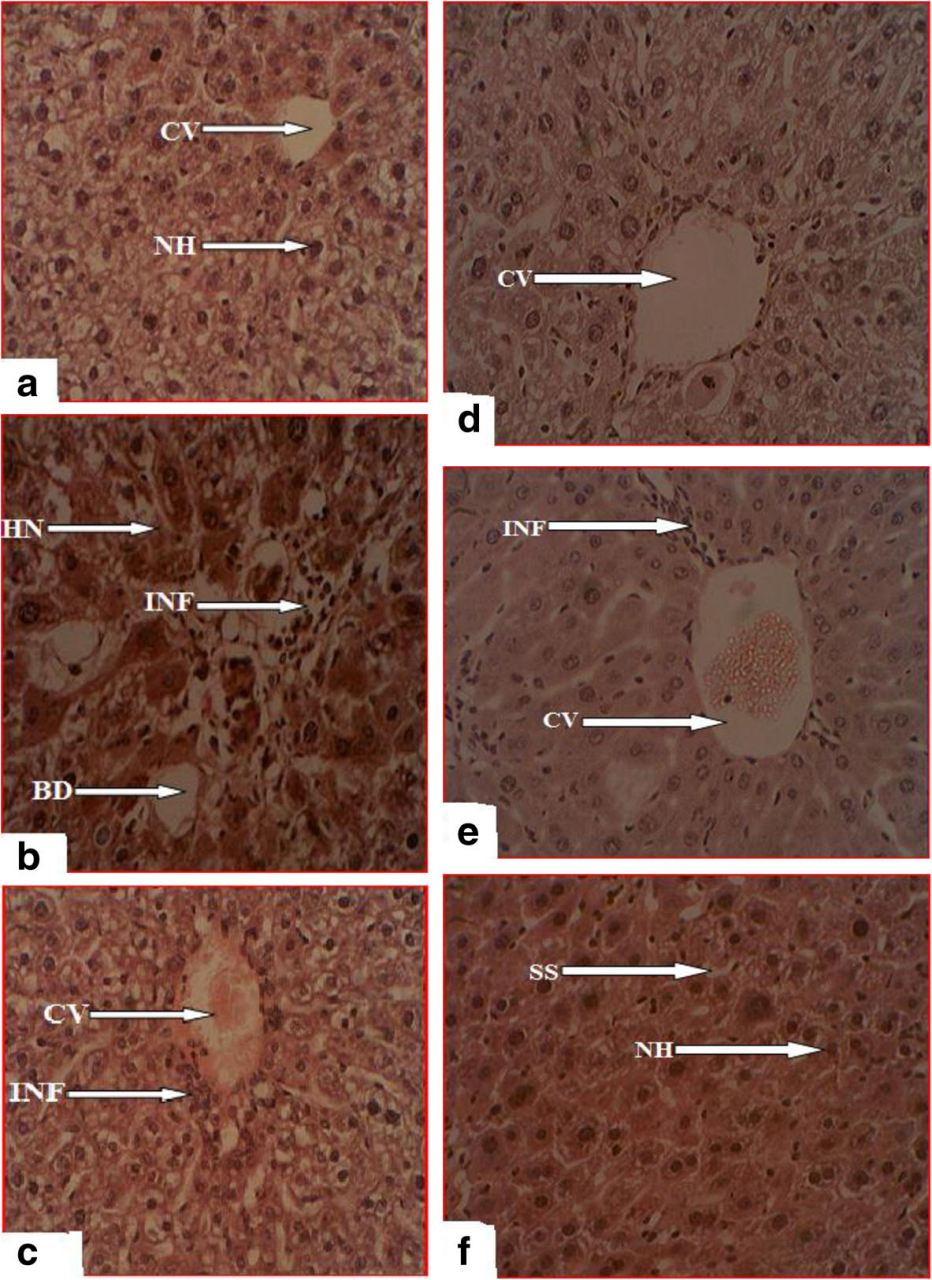
**a** Light micrograph of liver of normal mice (H&E). **b** Light micrograph of liver of CCl_4_ intoxicated mice (H&E). **c** Light micrographs of liver of silymarin administered mice (H&E). **d** Light micrograph of liver of EAF administered mice (H&E). **e** Light micrograph of liver of 90% EAF administered mice (H&E). **f** Light micrograph of liver of EAF+ Me administered mice (H&E)

Histological specimens of liver for control, CCl_4_, silymarin (100 mg/kg), EAF, partially purified EAF (90% EAF and EAF + Me) are shown in Fig. 5a-f. Mice administered with CCl_4_ showed extensive hepatocyte necrosis, severe fatty changes, sinusoidal congestion and lymphocytic infiltration as shown in Fig. 5b. VCME demonstrated moderate fatty changes accompanied by mild necrosis and infiltration. Liver sections of ethyl acetate and partially purified EAF administered mice showed significant recovery from necrosis, fatty changes, sinusoid congestion and lymphocytic infiltrations which is comparable to normal and in correlation with the biochemical tests.

### Effects of *Viola canescens* extract on living conditions of mice

General observations during the study period showed that CCl_4_ intoxicated mice exhibited anorexia, fur in a mess, poor activity and weight loss. These effects were reversed to a great extent by administration of *Viola canescens*.

## Discussion

The natural antioxidants reduce oxidative stress mediated damage and help prevent hepatotoxicity, carcinogenesis, mutagenesis and aging due to their radical termination potential. Moreover *Viola canescens* is enriched with phenols and flavonoids which are strong antioxidants [51, 52]. Oxidative stress is involved in a number of hepatic disorders that is one of the serious health problems across the globe [53]. Natural antioxidants eliminate oxidative stress caused by CCl_4_ and other hepatotoxicant [54]. The available synthetic antioxidants have some serious adverse reactions. The quest to discover natural antioxidants which are cost effective and have no/few adverse effects has become a challenge for scientists over the last decades. The current study involves investigation of total phenols and flavonoid contents, and the possible mechanism of antioxidant and hepatoprotective activity of *Viola canescens.*

DPPH and hydrogen peroxide radical scavenging assays are important tools for the assessment of antioxidant potential of extracts [55]. In DPPH assay, radical scavenging potential of *V. canescens* extracts may be attributed to a direct role in trapping free radicals by donating electron or hydrogen atom. Moreover, hydrogen peroxide is involved in generation of hydroxyl radicals, which cause further damage to the cells [56]. Therefore, it is important to search for more effective antioxidant compounds which exhibit good radical termination potential for ROS.

Carbon tetrachloride is bioactivated by cytochrome p450 enzymes resulting in the formation of free radicals that attack polyunsaturated fatty acids to generate peroxy and alkoxy radicals that, in turn, forms highly reactive lipid peroxides. The lipid peroxide formation cause loss of cell membrane integrity, leakage of enzymes, DNA damage, and hepatocyte necrosis [57]. Hepatocellular damage causes the leakage of liver biomarkers into serum. Enhanced ALT level shows loss of functional integrity of hepatocytes [58]. CCl_4_ also impairs bile flow with consequent increase in ALP and bilirubin level which are excreted through bile. VCME and its fractions restored the ALP and bilirubin level by membrane stabilization and prevention of biliary dysfunction. CCl_4_ also affect the protein synthesis in liver leading to decrease serum protein levels. VCME protects liver and restores its synthetic and metabolic function.

Catalase and SOD are the key antioxidant enzymes which play a major role in oxidative damage against oxidative stress induced by free radicals. The current study showed that carbon tetrachloride administration in mice results in decrease activities of CAT and SOD which is in corroboration with other investigations [59, 60]. CCl_4_ cause lipid peroxidation and increases the level MDA in hepatocytes. MDA, the secondary product of the lipid peroxidation, is an important indicator of tissue damages [61]. Administration of solvent extracts of *V. canescens* markedly decreased the MDA content near to normal as was revealed by other plant extracts [59, 60].

Lipid peroxidation product react with DNA to form mutagenic pirimedopurinone adduct of deoxyguanosine (M1G). Free radicals attack nucleic acids and cause oxidative damage to DNA and chromosomal alteration. In the current study, CCl_4_ degraded the DNA of mice liver tissue by free radicals formation [59, 62]. Co-administration of the solvent extracts appreciably reduced the DNA damage as shown by bands pattern in DNA ladder assay. Similar results were obtained in another study on the nephroprotective effects of Kombucha tea against CCl_4_ induced oxidative stress in rats [63].

Phenobarbital is mainly metabolised by cytochrome p450 enzyme system of the liver. An agent which inhibit cytochrome p450 enzyme will increase duration of phenobarbital induced sleeping time (PST) and vice versa. In case of CCl_4_ induced hepatotoxicity, prolongation of PST occurs due to destruction of the enzyme system. In the current study the shortening of PST observed after administration of VCME and its fractions show the ability of extract to improve metabolic function of liver.

RBC membrane is analogous to lysosomal membrane. Therefore, plant extracts which stabilize membrane of lysosomes means that it diminishes the leakage of lysosomal enzymes from activated neutrophils into surrounding tissue. NSAIDs may either inhibit lysosomal enzymes or stabilize the lysosomal membrane [47]. In a similar way RBC membrane is stabilized by VCME and EAF. Therefore, *Viola canescence* provides significant hepatoprotection by stabilization of hepatocyte membranes and limits the release of transaminase into the serum.

In toxicological experiments, comparison of changes in organ weight of animals is considered a sensitive indicator of drug toxicity [64, 65]. A significant difference in body weight between CCl_4_ intoxicated animals and extract administered groups was observed. The loss in body weight was significantly reduced by administration of EAF and partially purified EAF (90% EAF and 80% EAF + 20% Me) as compare to silymarin (*p* > 0.05). Similarly, the administration of EAF and partially purified EAF resulted in decrease of liver weight as compare to silymarin (*p* > 0.05).

The histological examination of liver specimens strongly supports the protective effect of *Viola canescens* solvent extracts. CCl_4_ administration resulted in fatty changes, sinusoidal congestion and piecemeal necrosis with loss of cellular archeitecture. The oral administration of EAF and partially purified EAF showed remarkable restoration of normal histological pattern of liver having optimum results as compared to silymarin.

## Conclusion

It may be concluded from the current study that hepatoprotective activity of *Viola canescens* is likely due to free radical scavenging, membrane stabilization potential and protection of endogenous antioxidant defense system. Further investigation to isolate and purify the active constituents responsible for hepatoprotection needs to be carried out. Findings of this study are expected to play a vital role in the development of new and effective hepatoprotective remedy.

## Abbreviations

ALP: Alkaline phosphatase
ALT: Alanine transaminase
AQF: Aqueous fraction
BD: Ballooning degeneration
CAT: Catalase
CCl_4_: Carbon tetrachloride
CF: Chloroform fraction
CV: Central venule
EAF: Ethyl acetate fraction
HN: Hepatocellular necrosis
INF: Cellular infiltration
MDA: Malondialdehyde
NH: Normal hepatocyte
NHF: *n*-hexane fraction
SOD: Superoxide dismutase
SS: Sinusoids
T.B: Total bilirubin
T.P: Total protein
TCA: Trichloroacetic acid
TE buffer: Tris-EDTA buffer
VCME: *Viola canescens* methanolic extract
